# PD1/PD-L1 inhibition as a potential radiosensitizer in head and neck squamous cell carcinoma: a case report

**DOI:** 10.1186/s40425-016-0187-0

**Published:** 2016-11-15

**Authors:** Misako Nagasaka, Mark Zaki, Harold Kim, S. Naweed Raza, George Yoo, Ho-sheng Lin, Ammar Sukari

**Affiliations:** 1 Department of Oncology, Barbara Ann Karmanos Cancer Institute, Wayne State University School of Medicine, Detroit, MI USA; 2 Division of Radiation Oncology, Department of Oncology, Barbara Ann Karmanos Cancer Center, Wayne State University School of Medicine, Detroit, MI USA; 3 Department of Otolaryngology-Head and Neck Surgery, Barbara Ann Karmanos Cancer Institute, Wayne State University School of Medicine, Detroit, MI USA

**Keywords:** PD1/PDL1 inhibitor, Oral cancer, Radiation therapy

## Abstract

**Background:**

Immunotherapy targeting the checkpoint PD1 (programmed cell death protein 1) or PDL1 (programmed death ligand 1) has led to advances in the treatment of melanoma and non-small cell lung cancer (NSCLC). The use of such therapies has also been introduced into the treatment of other malignancies, including head and neck cancer. The combined effects of checkpoint inhibitors and anti-PD1(L1) antibodies and radiation therapy have not yet been sufficiently investigated.

**Case presentation:**

We report a case of locally relapsed non-resectable oral cavity squamous cell carcinoma, with excellent local control after pembrolizumab (MK3475) followed by radiotherapy.

**Conclusion:**

T cell activation induced by checkpoint inhibition may dramatically improve tumor response to radiation. More data are needed to identify the toxicity and efficacy of sequential or concurrent checkpoint inhibitors and radiotherapy.

## Background

The development of immunotherapy targeting the PD1/PDL1 checkpoint inhibition pathway represents considerable progress in the treatment of many cancer types. Pembrolizumab is a humanized monoclonal antibody that blocks the interaction of PD-1 with its ligands, PD-L1 and PD-L2. It is FDA approved for the treatment of melanoma and NSCLC and was recently granted accelerated approval for the treatment of recurrent or metastatic head and neck squamous cell carcinoma in patients with disease progression on or after platinum-containing chemotherapy [1]. Little is known regarding the effects of radiation following PD1 inhibition. We report a case of a patient who experienced excellent local control with immunotherapy followed by radiation therapy for relapsed oral cavity cancer.

## Case presentation

**Fig. 1:**
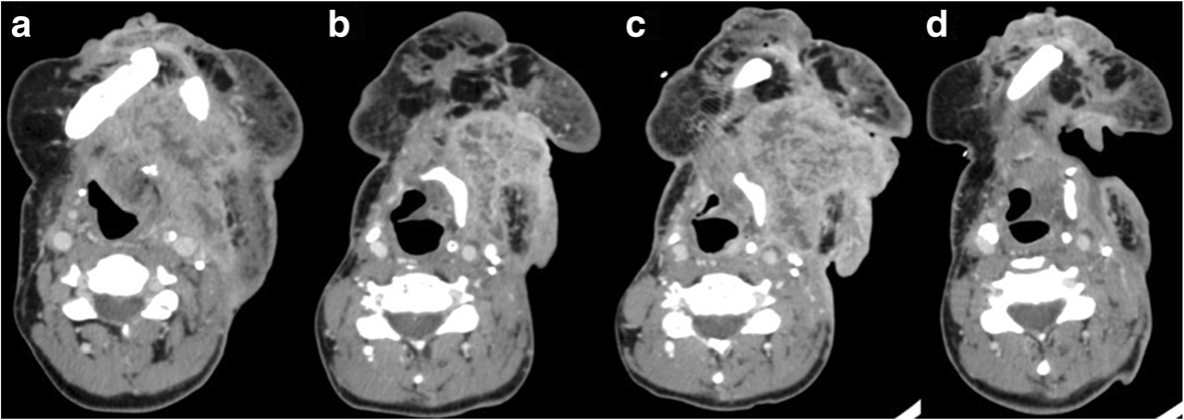
Change in largest dimensions of neck mass on CT scans over treatment period. **a** Prior to pembrolizumab. 8.8 × 5.9 cm. **b** Best response to pembrolizumab. 6 × 4 cm. **c** Progression on pembrolizumab. 7.1 × 7.2 cm. **d** Post radiation 5.9 × 3.4 cm

**Fig. 2:**
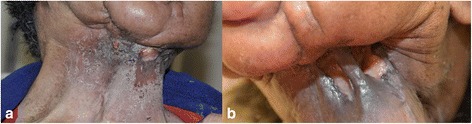
Appearance of neck mass post pembrolizumab and radiation therapy. **a** Local control was achieved after 6 cycles of single agent pembrolizumab therapy. **b** The bleeding mass resolved after radiation therapy

A 66 year old woman with floor of mouth squamous cell carcinoma (SCC) presented to our institution after her second relapse. Originally diagnosed in 2006, she had undergone a composite resection with a flap reconstruction and bilateral neck dissections followed by post-surgical adjuvant radiotherapy for stage IVa (T4aN0M0) disease. Immunohistochemistry (IHC) staining for p16 was negative. In May of 2009, a resectable locoregional recurrence was detected and consequently treated with a composite resection utilizing a pectoralis flap reconstruction. In November of 2013, she presented with a second non-resectable locoregional relapse. She received carboplatin and paclitaxel for 4 cycles with a partial response (PR) after 2 cycles. The patient subsequently developed regional progression and was treated with weekly methotrexate and cetuximab and she achieved stable disease (SD) for 6 months. Later, she progressed locally and was enrolled into a trial utilizing single agent pembrolizumab. She had SD for 6 cycles (Fig. 1), and then suffered from local progression with a significant increase in the size of her neck mass, with painful ulceration and bleeding. Pembrolizumab was therefore discontinued. At this time restaging studies revealed no evidence of distant metastasis. She required multiple transfusions secondary to tumor hemorrhage and as a result was treated palliatively with radiation therapy to a total dose of 30 Gy directed at the mass. The patient experienced an excellent clinical response. Bleeding had resolved (Fig. 2) and her pain had greatly improved. A significant radiographic response was also noted on computed tomography (CT) scan, with tumor dimensions decreasing by 60 %, from 7.1 × 7.2 cm pre-radiation, to 5.9 × 3.4 cm, 6 weeks post-radiation.

## Discussion

### Pembrolizumab in head and neck cancer

The strongest available data for checkpoint inhibitors in head and neck SCC are from an expansion cohort of a phase Ib study (KEYNOTE-012), utilizing pembrolizumab in the recurrent/metastatic setting (Table 1). One hundred and ninety-two patients were enrolled. Confirmed objective response rate (ORR) was 17.7 % (95 % CI, 12.6–23.9 %; 7 complete responses [CRs], 27 PRs). Thirty three (17 %) patients achieved stable disease. ORR was seen in 21.9 % (95 % CI, 12.5–34.0 %) of HPV (human papilloma virus) positive and in 15.9 % (95 % CI, 10.0–23.4 %) of HPV negative patients. The median overall survival (OS) was 8.5 months (95 % CI, 6.5–10.5). These were patients who were heavily pretreated and a majority of them had more than two lines of previous therapy. Treatment-related adverse events (TRAEs) occurred in 122 (64 %) patients; 23 (12 %) patients had a grade 3–4 TRAE [2].

**Table 1:** Ongoing trials on PD1 inhibitors in HNSCC

Abbreviated Trial Name/NCT#	Phase	Agent(s)	Study population	Findings/Expected Primary Endpoint	Safety
KEYNOTE-012/NCT01848834 Data updated from ASCO 2016	Ib	Pembrolizumab	Recurrent/metastatic HNSCC	ORR 17.7 % (95 % CI, 12.6–23.9 %; 7 CRs, 27 PRs). HPV+ 21.9 %, HPV- 15.9 %. Median OS 8.5 mo (95 % CI, 6.5–10.5).	Grade 3–4; 12 % No treatment related deaths
KEYNOTE-055/NCT02255097 Presented ASCO 2016	II	Pembrolizumab	Recurrent/metastatic HNSCC, progressed on platinum and cetuximab	ORR 18 % (95%CI 9–31); HPV+ 22 %, HPV- 16 % SD 18 %	Grade 3–5; 20 %
KEYNOTE-040/NCT02252042 Ongoing	III	Pembrolizumab VS Chemotherapy (methotrexate, docetaxel or cetuximab)	Recurrent/metastatic HNSCC	PFS OS	
KEYNOTE-048/NCT02358031 Ongoing	III	Pembrolizumab VS Pembro + cis/carbo + 5FU VS Cetuximab + cis/carbo + 5FU	First line treatment for recurrent/metastatic HNSCC	PFS	
CheckMate141/NCT02105636 Presented AACR 2016	III	Nivolumab VS Chemo (methotrexate, docetaxel or cetuximab)	Recurrent/metastatic HNSCC	1 year OS; nivo 36 %, chemo 16.6 % Median OS; nivo 7.5 mon, chemo 5.1 months	

### Radiation therapy and immunotherapy

The effects of radiation following PD1 inhibition are unknown. Current data come from the concurrent administration of immune checkpoint inhibitors with radiotherapy. Radiation is thought to enhance antitumor immune responses by causing inflammatory cell death, major histocompatibility complex (MHC) I upregulation, and release of antigens that are taken up by dendritic cells [3]. Mouse models have shown increased PD-L1 expression in tumors following irradiation [4]. The abscopal effect; or the phenomenon in which tumor regression occurs at sites distant from the site of radiation, has been documented in melanoma and NSCLC patients who underwent radiation with ipilimumab, a CTLA-4 (cytotoxic T-lymphocyte-associated protein 4) checkpoint inhibitors [5, 6]. This further supports the concept of synergistic activity between checkpoint inhibitors and radiation.

Identifying the most beneficial timing for combined radiotherapy and immunotherapy remains a challenge. If radiation is given prior to, or concurrently with immunotherapy, immunotherapy may be more effective with tumor specific antigens originally generated by radiotherapy. On the other hand, if immunotherapy is delivered before radiotherapy, the active immune microenvironment may maximize radiation efficacy [7]. In the present case, radiotherapy was given immediately following discontinuation of pembrolizumab in an attempt to control bleeding. The excellent response seen in the present case may be attributed from the synergistic effect of pembrolizumab.

One possible disadvantage of the concurrent administration of checkpoint inhibitors and radiation is the potential for added toxicities. In the present case, it is probable that the risk of adverse events (AE) was mitigated by the sequential delivery of therapy. In an analysis of 29 unresectable/metastatic melanoma patients who underwent radiation while receiving ipilimumab, the authors concluded that concurrent therapy was not associated with higher than expected rates of AEs, nor did it invalidate the palliative effects of radiation or survival benefits from ipilimumab [8, 9].

Several clinical trials are evaluating combined radiotherapy and checkpoint inhibitors in head and neck SCC (Table 2). The phase Ib study of cetuximab, ipilimumab and intensity modulated radiation therapy (IMRT) in stage III-IVa HPV+ oropharyngeal SCC (NCT01935921) and the phase II study of concurrent versus sequential pembrolizumab, cisplatin and IMRT in stage III-IVb head and neck SCC are currently accruing patients (NCT0277385).

**Table 2:** Ongoing studies on PD1 inhibitors and radiation therapy in HNSCC

Abbreviated Trial Name/NCT#	Phase	Agent(s)	Study population	Expected Primary Endpoint
NCT01935921	Ib	Cetuximab, ipilimumab and IMRT	stage III-IVa HPV+ OPSCC	Dose limiting toxicities (DLT)
RTOG 3504 NCT02764593	III w/phase I lead in	Nivolumab and cisplatin CRT	stage III-IV, intermediate to high risk HNSCC	DLT for phase I
NCT02777385	II	Concurrent vs sequential pembro, cisplatin and IMRT	stage III-IVb HNSCC	1 year PFS 1 year failure rate Acute toxicity rates
HN003 NCT02775812	I	Adjuvant pembro, cisplatin and IMRT	high risk stage III-IV HSNCC	DLT
NCT02641093	II	Adjuvant pembro, cisplatin and IMRT	high risk stage III-IV HSNCC	Treatment related adverse events (TRAE) Disease free survival (DFS)

## Conclusion

As we await further data, a trial of radiation following immunotherapy could be considered for disease control in selected patients.

## Abbreviation

CR: Complete response
CT: Computed tomography
HPV: Human papilloma virus
IMRT: Intensity modulated radiation therapy
MHC: Major histocompatibility complex
NSCLC: Non-small cell lung cancer
ORR: Objective response rate
OS: Overall survival
PD-1: Programmed cell death protein 1
PD-L1: Programmed death ligand 1
PR: Partial response
SCC: Squamous cell carcinoma
SD: Stable disease
TRAE: Treatment-related adverse events
